# TYPES OF SOCIAL NETWORKS AND THEIR ASSOCIATION WITH MOBILITY AND DISABILITY IN LATE LIFE

**DOI:** 10.1093/geroni/igz038.635

**Published:** 2019-11-08

**Authors:** Talha Ali, Michael Elliott, Toni C Antonucci, Belinda Needham, Jonathan Zelner, Carlos Mendes de Leon

**Affiliations:** University of Michigan, Ann Arbor, Michigan, United States

## Abstract

Social networks are critical in maintaining late-life functional health, but, previous studies have focused on isolated dimensions of social networks. We examined whether network types, representing multiple interrelated network characteristics, are associated with mobility and disability among older adults in America. Data are from the National Social Life, Health, and Aging Project, a nationally representative study of 3,005 adults aged 57-85 years at baseline (2005-2006). In a previous analysis, five social network types were derived at baseline, based on nine observed network characteristics. Functional outcomes were examined during two follow-up waves in 2010-2011 and 2015-2016. Mobility-related function was assessed as the time (in seconds) to complete a 6-meter walk. Disability was defined as experiencing any difficulty in performing one of six activities of daily living (ADLs). We estimated the effect of network types on risk of ADL disability onset using logistic regression, and on mobility using generalized linear mixed models. Social network type was associated with mobility over time, such that older adults in the “restricted” network had significantly slower walking times than those in the “diverse” network. There was no association between network types and risk of disability onset in the primary analysis. However, sensitivity analyses showed a protective effect of the “partner-centered” network on a 5-year, but not a 10-year, risk of disability onset. Network types can elucidate older adults’ varied interpersonal and caregiving networks, and identify adults at risk of being socially isolated. However, the utility of network types in predicting late-life functional health may be limited.

